# Touchless monitoring of neonatal activity: a multi-center study

**DOI:** 10.1038/s41390-025-04294-5

**Published:** 2025-07-24

**Authors:** Paul S. Addison, Mridula Gunturi, Dean Montgomery, Rangasamy Ramanathan, Manoj A. Biniwale, Dale Gerstmann, Jeffrey Clemmer, Rena Nelson

**Affiliations:** 1 Research and Development, Acute Care & Monitoring, Medtronic, Technopole Centre, Edinburgh, UK; 2 Department of Pediatrics, Cedars Sinai Guerin Children’s, Cedars Sinai Medical Center, Los Angeles, CA USA; 3 Timpanogos Regional Hospital, Orem, UT USA

## Abstract

**Background:**

Neonatal activity level is an important physiological parameter linked to lethargic response to over sedation, agitation associated with under-titration of pain medication or seizures and serves as an early indicator of disease onset.

**Methods:**

Activity was monitored continuously using a non-contact (“touchless”) technology based on a depth sensing camera in 61 neonates from two sites (*N* = 32 ‘LA’ / 29 ‘Utah’). Gestational age, mean (SD): 34.1 (3.3) weeks Utah, 34.7 (3.8) weeks LA. Time after birth 2.5 (2.5) weeks Utah, 1.6, (1.9) weeks LA. This was compared to manual observation of motion using two main random forest machine learning analyses: [1] combined data set leave-one-out cross-validation (LOOCV) on a per-neonate basis and [2] inter-site analysis.

**Results:**

Combined analysis: mean [CI] sensitivity, specificity, and corresponding area under ROC curve for the test sets are 93.8% [92.3, 95.3], 92.2% [90.0, 94.3], and 98.4% [97.8, 99.0], respectively. Inter-site analysis: training using LA data and testing on Utah data resulted in corresponding results of 94.2% [92.0, 96.5], 81.5% [76.2, 86.7], 97.6% [96.5, 98.7], respectively. Utah training and LA testing produced 95.1% [93.3, 96.9], 91.9%, [88.9, 94.9], 98.9% [98.5, 99.4].

**Conclusions:**

Touchless monitoring can provide the basis for quantitative, continuous monitoring of neonatal activity.

**Impact:**

- We investigated the feasibility of non-contact (‘touchless’) monitoring of neonatal activity based on depth sensing technology.
- Touchless monitoring offers a sensor and wire-free solution for monitoring activity.
- The findings support the need for further investigation of the method to determine associations with the measured activity and clinical outcomes.

## Introduction

The level of activity of a neonate is an important physiological parameter in the neonatal intensive care unit (NICU). Pain originating from diagnostic or therapeutic interventions, or directly through a disease process is a major cause of pathological activity in neonates. To manage this pain and prevent any long-lasting physiological or neurodevelopmental effects, appropriate sedatives and analgesics are administered.^1^ However, determining the correct treatment option requires a clinical evaluation of pain. A number of manual pain and sedation scales are available^2^ including the Neonatal Pain, Agitation and Sedation Scale (N-PASS).^3,4^ N-PASS includes the manual assessment of activity as part of the score to determine both lethargy, associated with oversedation, or restlessness, associated with undersedation and pain. In addition to oversedation, neonatal lethargy may be a result of metabolic derangements, hypoxic-ischemic encephalopathy, infection/sepsis, inborn errors of metabolism, intraventricular hemorrhage and/or hyperbilirubinemia.^5^ Lethargic underactivity is the most frequent clinical sign associated with the onset of sepsis when considered among a range of other clinical signs including grunting, abdominal distension, increased prefeed aspirates, tachycardia, temperature instability and chest retractions.^6^ This underscores the significance of recognizing reduced activity when diagnosing diseases. Additionally, neonatal seizures can lead to pathological activity including involuntary jerking movements and can be caused by various conditions including hypoxic ischemic encephalopathy, infarction or intracranial hemorrhage, intracranial infections, brain malformations, and genetic or metabolic disorders.^7^

Neonatal activity, in the form of limb motion or gross movement of the torso, is also a major confounder for clinical monitoring devices where motion noise can significantly impact the quality of physiological signals. Affected signals may include the electrocardiogram (ECG), the transthoracic impedance (TTI) signal, pulse oximeter measurements and the photoplethysmogram (PPG), which are used to derive heart rate, respiratory activity (including respiratory rate and apnea) and saturation levels. These form the principal components for apnea-bradycardia-desaturation (ABD) monitoring in the NICU.^8,9^ Motion artifact often contaminates all these signals^10^ and is a challenge for robust, accurate and continuous neonatal monitoring. It is well known that the high prevalence of noise and high false alarm rates is the reason that respiratory signals are still largely disregarded in the NICU.^11^

These aspects of neonatal activity are the driving force behind our current research aimed at developing a robust and continuous, non-contact neonatal activity monitor. We hypothesized that a novel depth sensing camera system combined with a random forest machine learning model could provide a non-contact “touchless” method of monitoring neonatal activity. We compared the activity detected by this system with a manual annotation of the ground truth using data from two NICUs. We also explored how different factors, such as the position of the camera, the type of bed, gestational age and weight, affect the performance of our system.

## Methods

### Data acquisition and processing

Data was collected from a cohort of neonates from two sites: the NICU at Los Angeles General Medical Center, Los Angeles, CA (the ‘LA Data’), a 39-bed level III care facility and the NICU at the Timpanogos Regional Hospital, Orem, UT (the ‘Utah Data’) a 24-bed facility also offering level III care. More site details are provided in the Supplementary Material. Approved informed consent was obtained from parents for each participant covering the essential information stated in the protocol, as required elements according to 21 CFR 812.150 for a non-significant risk medical device investigation. The study was approved by the local institutional review board at each site. All neonates receiving care in a NICU were eligible for participation. Exclusion criteria included patients receiving high-frequency ventilation support and those contra-indicated for any monitoring device required for study participation per investigator discretion. In this preliminary pilot study we aimed to collect data from a convenience sample of neonatal subjects for algorithm development. As such the study is not statistically powered. Study results were used to assess feasibility of the technology and may also be used to gather information required to calculate sample sizes for future studies. Sixty subjects were specified for data analysis and algorithm development. The neonates exhibited a range of medical conditions. These are provided in Table S1 in the Supplementary Material. We did not test model performance with respect to medical condition as the data available was insufficient to perform a meaningful statistical analysis.

**Fig. 1:**
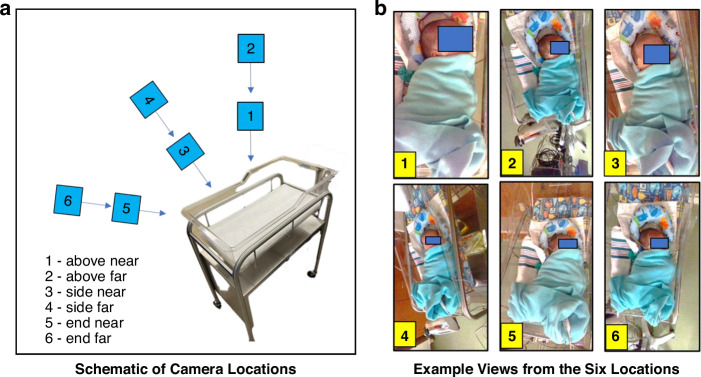
Camera locations around the neonatal bed with associated example views. **a** Schematic of Camera Locations. **b** Example Views from the Six Locations

Depth information was acquired at 30 fps from the scene using an Intel RealSense^TM^ D415 camera mounted on a tripod. This device captures the distance (depth) from the camera to all objects in its field of view and has been shown to be able to accurately monitor respiratory activity in adults.^12–16^ The research staff at the sites were asked to place the camera at six distinct locations relative to the incubator, bassinet or crib according to the schematic shown in Fig. 1a and record data for five minutes at each location. This was done in sequence using a single camera moved to each location in turn by the research staff. Two captures—one ‘near’ and one ‘far’—were taken above (locations 1 & 2), at the side (locations 3 & 4) and at the end (locations 5 & 6) of the bed. Figure 1b shows an example set of still images from the associated RGB feed taken at each of the six locations. The site staff were allowed to choose the exact position and asked to capture data within a distance of 800 mm from the camera to the neonate. Locations 3 and 4 could be captured at either side of the bed and locations 5 & 6 could be captured either at the head-end or foot-end of the bed and the choice was left to the discretion of the staff. We also did not specify the timing of data capture to coincide with specific clinical procedures or events. Data was collected when convenient for the staff to do so within their workflow. (Fig. S1 in the Supplementary Material contains the times when video acquisitions were made during the day at both sites).

**Fig. 2:**
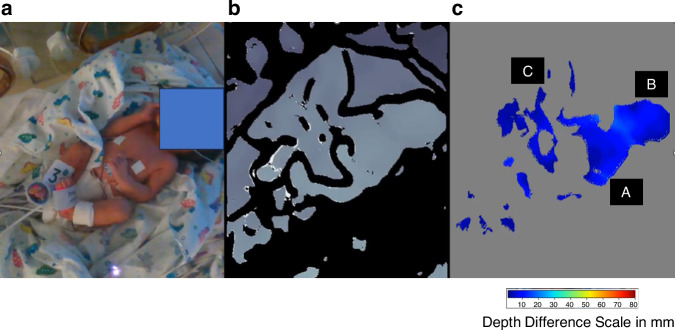
Example RGB, depth and depth difference captures of neonatal motion. **a** RGB Image of Neonate **b** the corresponding Depth Image **c** the corresponding Depth Difference Plot indicating Motion on the Upper Torso (**a**) and Head (**b**) together with associated Sheet Movement (**c**). (These are stills from the video in the Supplementary Material).

The acquired depth data was processed to provide depth difference frames prior to motion analysis. This was achieved by computing characteristic changes in depth between frames at 1-second intervals. Figure 2a–c contain an example RGB image capture of a neonate, together with the corresponding depth image and the depth difference calculated over the previous second. The depth difference was generated by averaging 10 consecutive frames at the beginning and the end of each second of the depth video stream and taking the difference between each of these temporal averages. Motion may be detected in these depth difference frames as non-zero values depicted by the color patches in Fig. 2c. An example of a video sequence of the depth difference values over time can be found in the Supplementary Material associated with this manuscript. The raw depth difference frames were pre-processed to remove noise as follows: [1] prior to taking the difference the raw depth frames above the 87^th^ percentile and lower than the 3^rd^ percentile in the scene were removed (as it was observed from the data that camera noise primarily occurs at higher depths, hence a wider upper depth percentile limit of 87% was applied); [2] spatial median filters of kernel size 5×5 and 3×3 were applied sequentially to the depth difference frames to remove small pixel regions containing unusually high or low depth values and thus smooth out the regions of motion; [3] high depth difference values of >150 mm were removed from the frame to ensure that only depth differences corresponding to physiologically possible neonatal motion were included; [4] small regions of valid depth differences (with area < 40 pixels), considered too small to be valid neonatal motion, were removed. A flow chart of the pre-processing steps is provided in Fig. S2. Finally, sixteen time-variant features were extracted from the depth and depth difference frames for input into the random forest classifier. The features were designed to represent various motion characteristics including the spatial scale and magnitude of motion, as well as accounting for noise. A more detailed description of the features is provided in Table S2 in the Supplementary Material. Table S3 provides a description of the parameters tested in the optimization of the preprocessing parameters and corresponding ranges of parameter values tested during optimization of the model.

### The random forest classifier

The model used for motion detection is a random forest binary classifier, an ensemble of decision tree models.^17^ Each decision tree is trained on a randomly drawn bootstrap sample from the data (sample drawn with replacement); to further increase diversity of the model, a random subset of all features is used to train each tree.^18^ The final class probability of the model (the probability of motion over a given second) is computed by finding the mean of the class probabilities of all decision trees in the random forest- this reduces overfitting and improves the generalization of the model.^19^ A random forest model was chosen over a more complex (e.g., deep learning) model for its simplicity, ease of interpretation, and robustness, especially when dealing with limited data or when interpretability is crucial. Random forests are computationally more efficient, less prone to overfitting and provide a means for measuring feature importance. Note that we followed the CAIR checklist guidelines, where applicable, in the reporting of the machine learning model and results.^20^

A grid-search method was used to optimize the denoising parameters (e.g. number of temporally-averaged depth frames, spatial median filter size, minimum area of connected pixel regions in the depth difference frame) and model hyperparameters (e.g. number of decision trees in the ensemble, maximum depth of each tree, maximum number of features used to train each tree). This optimization method aimed to maximize the mean square root of the area under the test receiver operator characteristic curves (ROC–AUCs) and was selected to minimize false positives and false negatives in the predicted motion signal of the model. Table S4 in the Supplementary Material contains the model hyperparameters, their values, and how each hyperparameter can be adjusted to mitigate overfitting. Note that the feature importances for the random forest model used to obtain the results presented below are provided in Table S5 in the Supplementary Material.

The classifier was trained and tested on a manually scored motion truth signal determined from visual observation of the corresponding RGB video stream. This was performed by one of the authors (MG—who has extensive experience in video labeling), where each RGB video sequence was assessed to determine whether any detectable motion was present in the scene. This was achieved by splitting the video into 1-s frames, then viewing the frames sequentially to determine if visually observable neonatal motion was present between frames. Note that no seizures were observed during the labeling process.

### Data analysis

We performed two main analyses—a leave-one-out cross validation across the combined dataset and an inter-site analysis. These are described as follows:

- **combined dataset leave-one-out cross-validation (LOOCV):** This was performed on a per-neonate basis and allowed for the inter-subject variability of the method across all the data to be characterized. Here, the labeled reference data from 59 neonates were used to train the model which was then tested on the data from the remaining ‘left-out’ neonatal data set (comprising the depth video captures for that neonate for each camera location). This process was repeated 60 times in order that the model was tested on all of the (up to) six positional test runs from each neonate. Training ROC curves were generated from the sensitivity (SENS) and specificity (SPEC) pairs associated with the training and an optimal class probability threshold was determined, defined as the point at which the Youden Index of the training results is maximized. The Youden Index is a statistical measure which combines SENS and SPEC into a single metric, used to evaluate the effectiveness of a diagnostic test: defined as YI = SENS + SPEC - 1 [31]. All performance statistics are reported in terms of mean value and confidence interval (CI).
  We performed secondary analyses of the LOOCV results with respect to various subgroups: [1] camera location, [2] gestational age, [3] weight and [4] bed type. Box plots of the sub-groupings were plotted and the Kruskal-Wallis statistical test to compare the medians of two or more independent groups was used to determine significant differences in the performance measures across the sub-groups.^21^
- **inter-site analysis:** a train-test-per-site switching (i.e. training on one site and testing on the other and vice versa). This allows for a check that we are not simply overfitting to a single site when training the model. The method of identifying the optimal class threshold and performance measures were the same as used in the LOOCV analysis and the same performance metrics were used to report the results.

## Results

Data was collected from 61 neonates, split approximately evenly between the two sites: *N* = 32 ‘LA Data’ and *N* = 29 ‘Utah Data’. Sex: 20 male / 9 female Utah; 17 male / 15 female LA. Gestational age: mean (SD): 34.1, (3.3) weeks Utah; 34.7, (3.8) weeks LA. Time after birth: 2.5, (2.5) weeks Utah; 1.6, (1.9) weeks LA. Weight: 2.48, (0.69) kg Utah; 2.40 (0.80) kg LA. Height: 45.5, (3.5) cm Utah; 45.0, (5.0) cm LA. Full demographic data is provided in Table 1.

**Table 1:** Demographic data for the Utah and LA data sets.

Utah subject ID num	Sex	Weight (kg)	Height (cm)	Gestational age (weeks)	Time after birth (weeks)	Bed type
**(a) Utah Data**						
1	M	2.11	42.9	35	9.0	Basinette
2	M	1.43	40.6	31	0.9	Closed incubator
3	M	1.75	41.8	31	1.1	Closed incubator
4	F	1.69	38.8	28	4.0	Closed incubator
5	M	3.66	49.5	36	1.6	Basinette
7	M	3.76	53.3	39	0.4	Open incubator
8	F	2.37	42.6	34	6.0	Basinette
9	F	2.33	44.7	37	2.9	Bassinette
10	M	1.71	44	35	0.3	Open incubator
13	M	3.08	49.5	39	0.7	Bassinette
14	M	1.75	43	31	2.4	Open incubator
17	F	2.74	45.2	34	1.6	Bassinette
18	M	1.69	44.5	32	1.0	Open incubator
19	M	2.26	44.3	32	3.4	Bassinette
20	M	2.87	48.3	38	0.1	Open incubator
21	F	2	39.5	28	7.6	Bassinette
22	M	2.6	46.7	37	1.0	Open incubator
24	M	3.89	49.5	31	6.6	Bassinette
25	M	3.21	49.6	34	3.3	Bassinette
26	F	1.89	41.6	28	7.3	Bassinette
27	M	3.24	51.6	37	2.1	Bassinette
28	M	2.39	46.3	34	0.9	Open incubator
29	M	2.01	44.2	32	1.7	Closed incubator
30	M	2.07	46.6	35	1.3	Open incubator
31	M	2.69	47.5	38	0.4	Open incubator
32	F	1.8	43.5	34	2.0	Bassinette
33	F	3.28	48	39	0.4	Open incubator
34	M	2.86	45.6	33	2.6	Bassinette
35	F	2.85	46.3	37	0.7	Bassinette
	**Mean**	2.48	45.5	34.1	2.5	
	**SD**	0.69	3.5	3.3	2.5	
**(b) LA Data**						
1	M	2.86	49	35	0.1	Closed incubator
2	M	2.84	49	39	0.4	Crib
3	M	2.58	45.5	34	0.4	Closed incubator
4	M	2.29	43	29	6.0	Crib
5	M	1.94	45	35	0.6	Closed incubator
6	F	1.72	44	35	0.9	Closed incubator
7	F	1.09	36	27	3.0	Closed incubator
8	M	2.5	47.5	35	1.6	Bassinette
9	M	1.86	45.5	31	4.0	Closed incubator
10	M	1.71	42.5	30	4.9	Closed incubator
11	F	2.85	40	38	0.9	Crib
12	M	2.65	48	31	7.6	Crib
13	M	3.23	53	38	0.3	Closed incubator
14	F	2.09	44	36	0.3	Closed incubator
15	F	2.13	45.5	36	0.4	Closed incubator
16	F	2.86	49	39	0.3	Closed incubator
17	M	0.89	34	32	0.7	Closed incubator
18	M	2.57	45.5	34	0.3	Closed incubator
19	M	2.77	46	36	1.1	Crib
20	M	2.22	42	37	1.9	Bassinette
21	F	1.77	44.5	35	0.7	Closed incubator
22	F	3.34	52	39	0.7	Crib
23	F	2.78	47	36	1.6	Bassinette
24	F	3.5	49	38	0.1	Closed incubator
25	M	3.63	53	40	1.3	Crib
26	F	1.83	41.5	34	1.9	Closed incubator
27	M	1.98	41	37	0.4	Closed incubator
28	F	3.92	50	37	0.6	Bassinette
29	M	1.05	36	26	1.0	Closed incubator
30	F	2.67	47	38	0.4	Bassinette
31	F	3.71	50	36	4.9	Bassinette
32	F	1	35	26	2.1	Closed incubator
	**Mean**	2.40	45.0	34.7	1.6	
	**SD**	0.80	5.0	3.8	1.9	

The LA neonates were in incubators (*N* = 19), bassinets (*N* = 6), or cribs (*N* = 7) as appropriate for their condition. Similarly, the Utah neonates were in incubators (*N* = 14) and bassinets (*N* = 15). At the LA site all incubators had their top covers in place, whereas only 4 of the Utah neonatal incubators had their top covers in place during the capture, with the remaining 10 being uncovered. Importantly, bassinets, cribs, and uncovered incubators allow a clear view of the neonate whereas the depth camera is required to sense through the plastic covering for the covered incubators. Bed types are provided in Table 1 alongside the demographic data. The neonates had various coverings. Of the sixty one neonates that participated in the study, 45 were swaddled, 7 had a loose blanket covering, 1 was partially covered by a loose blanket and 7 were uncovered with no sleepsuit (i.e., naked apart from diaper). Motion was observable through all coverings.

**Fig. 3:**
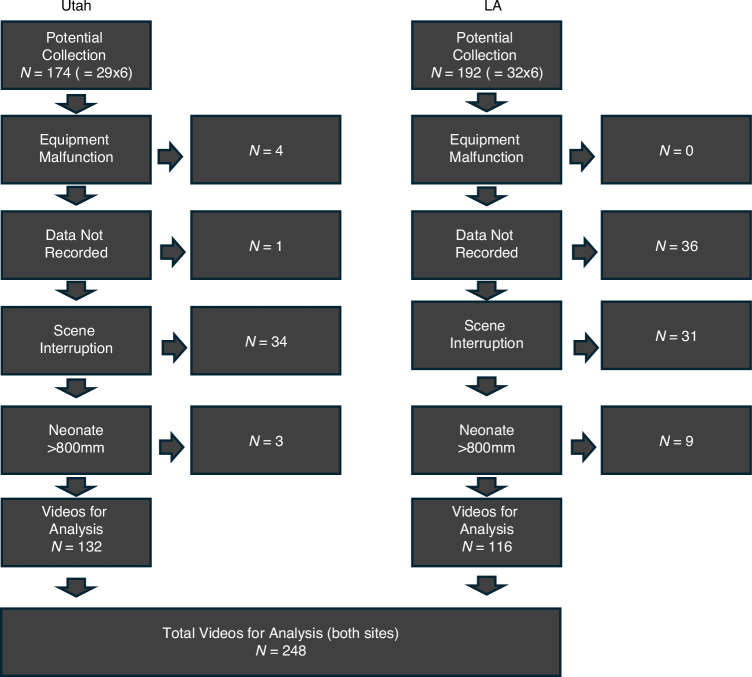
Data flow chart. Breakdown of the Utah and LA data captures. Out of a potential *N* = 366 test videos, *N* = 248 test videos were available for analysis.

It was not possible to collect at all camera locations (Fig. 1b) for each neonate due to various causes. At the Utah site, the camera arm malfunctioned during the depth data acquisition for two neonatal collections, hence, depth data for only 3 and 5 positions, respectively, was collected; and for one further neonate, depth data was inadvertently collected for only 5 positions. At the LA site, the data for positions 5 and 6 were not collected for 18 neonates due to time constraints towards the end of the study. Scenes that were interrupted by clinical staff obscuring the field of view were also discarded, reducing the data by 34 and 31 datasets from the Utah and LA data sets, respectively. (Note that this resulted in all six positional test runs for one neonate from the LA site being removed since they contained scene interruption by clinical staff, hence data sets from only 60 neonates were available for analysis.) In addition, the clinical staff were relatively free to place the camera in each position and some data was collected further than the specified maximum distance of 800 mm: these data sets were also discarded from the analysis. This reduced the data by 3 and 9 data sets, respectively at the Utah and LA sites. Thus, a total of 248 positional test-run acquisitions were available for analysis, each of around 5 min in length, corresponding to a total of 79,473 s of test data. This final data set was used without further processing (i.e., separate handling for extreme values or outliers). Figure 3 provides the full breakdown of the data available for analysis.

### Leave-one-out cross-validation

**Fig. 4:**
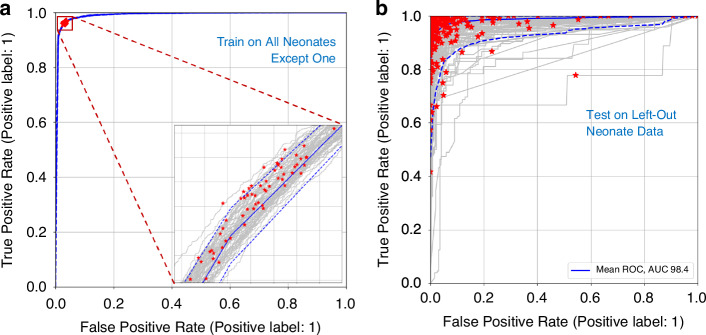
ROC curves for the leave-one-out cross validation analysis. Training ROC curves (**a**) and test ROC curves (**b**). The optimal points are indicated on each of the ROC curves by a red asterisk. The mean is shown as a solid blue line and the confidence intervals as dashed blue lines. (The upper confidence level in (**b**) is indecipherable as it is close to the axes).

The training ROC curves for the leave-one-out cross-validation are shown in Fig. 4a. The figure shows all training ROC curves with the optimal points indicated for each in the zoomed-in plot. We can see that the curves and optimal points on the curves are similar, as each aggregated set differs only by the remaining ‘left-out’ set. For the training, the mean value of the ROC-AUCs was 99.4% [99.4, 99.4] and the mean optimal SENS and SPEC was 96.3% [96.2, 96.3] and 96.7% [96.7, 96.8] respectively, where very tight limits of agreement are apparent in the training set. The optimal point on each training curve was used to determine the class probability threshold to test the remaining test data corresponding to the left-out neonate, where activity is predicted when class probability output from the model during training is greater than the threshold. The results are depicted in Fig. 4b. Note that we have included the full ROC curve in the plot for each test set, although the operating point for each (indicated by the red dots) is dictated by the threshold found in training. The mean (CI) of the SENS and SPEC and corresponding ROC-AUC for the test sets using the thresholds found in the training are 93.8% [92.3, 95.3], 92.2% [90.0,94.3], and 98.4% [97.8, 99.0], respectively.

### Subgroup analysis of the LOOCV results

**Fig. 5:**
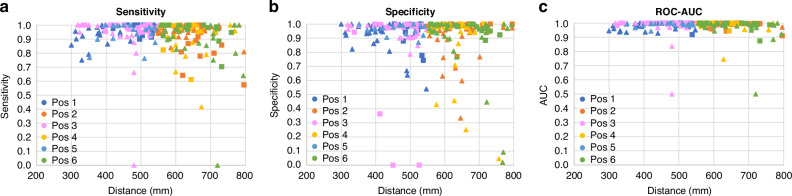
Sensitivity, specificity, and ROC-AUC for the leave-one-out cross-validation analysis for each camera location plotted against distance (square = LA Data. Triangle =  Utah data). **a** Sensitivity. **b** Specificity. **c** ROC-AUC.

**Fig. 6:**
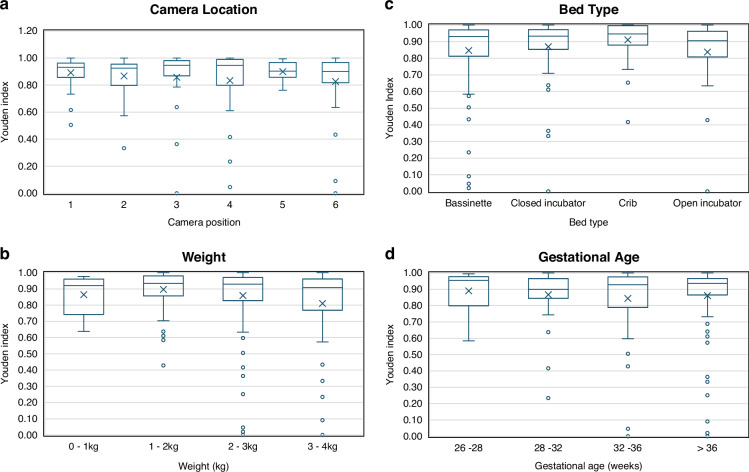
Youden index boxplots for the subgroup analysis. **a** Camera location. **b** Weight. **c** Bed type. **d** Gestational age.

The results were stratified according to camera positions 1 to 6 (as shown in Fig. 1). The individual SENS, SPEC, ROC-AUCs obtained for each test capture from the leave-one-out analysis reported above are plotted in Fig. 5. The groupings of the *near* (positions 1, 3, and 5) and *far* positions (2, 4 and 6) are also evident in the plot, illustrating the range of distances over which the clinical staff placed the camera. Table S6 in the Supplementary Material contains the stratified performance statistics for each camera position where it can be seen that high values of Youden Index are achieved (range 0.83 – 0.90). It can also be seen from the table that for each near/far pair the far value has a lower Youden Index. The associated box plots for the Youden Indexes for the six camera positions are provided in Fig. 6a. The corresponding box plots for the results stratification with respect to weight, bed type, and gestational age are provided in Fig. 6b–d. The Kruskal-Wallis test on each stratification subgroup found no significant intra-subgroup difference, (i.e., all *p* values > 0.05 as reported in Table S6).

### Inter-site analysis

The training ROC curves for the inter-site analysis are provided in Fig. S3a, b in the Supplementary Material for LA and Utah, respectively. The position of the optimal point found on the training ROC curve for each site is highlighted in the plots. The ROC-AUC was 99.8% and the optimal SENS and SPEC were 98.3% and 97.3%, respectively for the LA training data and correspondingly 99.2%, 96.6% and 95.6%, respectively for the Utah training data. The results from applying the training-derived class probability threshold to the test sets from each site are shown in Fig. S3c, d for the two sites. Training on the LA data and testing on the Utah data resulted in SENS, SPEC, ROC-AUC in terms of mean and CI of 94.2% [92.0, 96.5], 81.5% [76.2, 86.7], 97.6% [96.5, 98.7], respectively. Training on the Utah data and testing on the LA data produced 95.1% [93.3, 96.9], 91.9%, [88.9, 94.9], 98.9% [98.5, 99.4].

The results are provided in tabular form in Table S7 in the Supplementary Material.

## Discussion

The neonatal activity monitoring algorithm we have developed demonstrated excellent performance, for both an inter-site analysis and the leave-one-out cross validation on a per-neonate basis. The high sensitivities and specificities obtained, when comparing our activity algorithm output to a manually scored reference, indicate that it performs well at separating out active and non-active periods. The random forest model is simple to train and relatively robust across datasets. The work here has demonstrated that the model can detect neonatal movements of varying magnitude and spatial scale.

In other work by our group, for adults we place the camera at *1.1 m directly above the patient torso when monitoring adults* in order to optimize the ability to detect respiratory activity across the whole bed, i.e., as the patient moves around the bed, we can still view the torso while achieving good performance^22,23^ Neonates (and their beds) are much smaller, and this allows the camera to be brought closer. However, we currently do not know what position a final system might be optimally located at for performance and/or ergonomic considerations. It is for this reason that we tested at six positions comprising three orientations (above, side, and end) and distances (near and far, covering a range of depths up to 800 mm). We found no significant differences in the performance measures across the six camera positions when we stratified the results accordingly for the LOOCV analysis.

However, for the inter-site analysis, when training on the LA site and testing on the Utah site we noticed a markedly lower specificity than when Utah data was used in training and LA data in testing: that is, 81.5% compared to 92.2% for the similar sensitivities of 94.2% and 93.8%. On further inspection of the data, it was noticed that many of the poorer results were associated with the upper end of the collection limit (of 800 mm) and corresponded to the Utah neonatal set being noisier at the larger distances. To investigate this further, we reduced the maximum distance limit to 700 mm (down from the original 800 mm) and reran the analysis. This improved the specificity from 81.5% to 87.3% with very little change in the sensitivities (94.2% and 94.6%). This suggests that getting closer to the neonate may be a simple way to generate a marked improvement in performance. In summary, the inter-site analysis showed that the method was less generalizable between two sites, although this appears to be a function of distance from the camera to the neonate and the results improved when removing data acquired farther from the camera. This would indicate that, in practice, the training would require data from more sites.

We also performed a stratification analysis of the LOOCV results with respect to gestational age, weight, and bed type. Again, there were no significant differences found in the performance measures across these sub-groups. This may indicate that the null hypothesis is true and the method was robust to these factors or, alternatively, that further evidence is required to draw any conclusions. In summary, the proposed method achieved high accuracy in detecting motion from depth videos of neonates in different clinical settings.

As an additional comparator, at the suggestion of a reviewer, we performed a LOOCV analysis on each site separately to demonstrate how the model independently performs at each NICU without cross-site influences. Training and testing on the LA data alone achieved test performances of SENS, SPEC, ROC-AUC of 96.4%, 93.6%, and 99.2% respectively, and for the Utah data alone 92.1%, 91.8%, and 97.4% respectively. (Full results with confidence intervals are provided in Table S7 in the Supplementary Material.) Interestingly, these separate site performances are an improvement over that of the combined dataset analysis (93.8%, 92.2% and 98.4% respectively) for the LA dataset and poorer for the Utah data set, which would appear to confirm the noisier nature of the Utah data compared to the LA data.

**Fig. 7:**
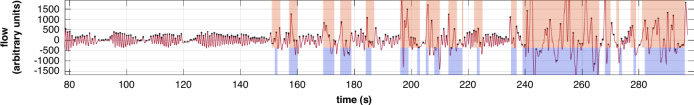
An example result of the predicted motion (red patches) and true (labeled) periods of motion (blue patches) superimposed onto the corresponding respiratory flow signal. The example shown corresponds to the first neonate in the Utah dataset (camera location 3).

Figure 7 provides an example of periods of motion predicted by the random forest model superimposed onto the respiratory signal also generated by the touchless system. We can observe that the activity flag coincides with recognizable motion noise on the respiratory signal. In practice the activity algorithm could be used to parse out motion noise from the good respiratory signal and provide an indication of the validity of the associated respiratory rate vital sign. This could lead to a number of strategies for dealing with this signal interference which could be employed to better aid the clinician in interpreting the data: these could include switching the algorithm to a more motion tolerant state, indicating on screen that motion is occurring and that the vital sign may not be reliable or, in extreme cases, ceasing to report the vital sign on screen.

We are currently engaging with hospital administrations and NICU staff both in the UK and in the US to assess the viability of introducing a quantitative activity parameter and how it will fit with workflows. As with the introduction of all new parameters, there is a learning curve and workflow fit that needs to be addressed. As part of our assessment of the activity measure, we will also undertake user interface design and perform outcome studies to demonstrate its utility. Ethical considerations will also need to be taken account of including unbiased accuracy across subgroup demographics with a view of maintaining health equity. We are at an early stage in the process of assessing the viability of this parameter and its implementation within clinical practice. Furthermore, implementing the proposed depth sensing activity system in practice would require significant computation to process the incoming data stream and perform the analysis in real time. We envisage that the system would work in a similar way to other monitoring devices such as the ECG or pulse oximeter. That is, it would not record the raw signals but rather be a continuous monitoring tool where the activity measure is computed in real time. The depth video data would likely be analyzed local to the patient due to bandwidth and/or privacy issues, perhaps on a bedside monitor or computer, i.e. on an edge computing device. Thus the raw video data would not need to be transferred to the cloud or elsewhere in the hospital. The activity measure output from this edge analysis could then be transferred to a local monitor, including displays embedded within incubators, or sent elsewhere, e.g. a server in the hospital or to the cloud for input into the EMR or other data storage system. This activity measure itself may be an indication of current activity, or may be further processed to provide a frequency of activity or intensity of activity. Further assessment of the final form of the activity measure and its clinical utility is ongoing.

Wearable sensors (e.g. accelerometer-based) offer an alternative modality for removing the requirement for wired connections, however, they must be comfortable for infants to wear without causing stress or skin irritation and they often need precise placement to reduce motion noise and optimize data acquisition.^24^ They also require sufficient battery life and, if used for the continuous monitoring of vital signs, constant wireless connectivity is essential.^25^ Touchless sensing has none of these drawbacks. An additional advantage of the touchless technology is that it utilizes a standard depth camera without the need for hardware modifications. The system is user-friendly, requiring no calibration and no attachment of sensors to the patients, making it ideal for use with these most vulnerable patients. Video cameras are already widely used in the NICU to strengthen parent-infant bonding and alleviate parental stress.^26,27^ This makes the NICU an ideal environment for integrating a depth-sensing camera system for activity monitoring. The camera used in the study, the Intel RealSense™ D415, provides RGB, IR, and depth modalities. It can live stream color video to parents or caregivers while using the depth modality for separate clinical evaluation of activity. Additionally, the depth camera system holds potential for further physiological monitoring in the NICU, such as tracking respiratory rate, tidal volume, respiratory patterns, and detecting apnea, as it has been demonstrated in adults.^12^

There are several limitations associated with the study. The manual labeling, which was undertaken by viewing the RGB video feed, may be subject to error in interpretation of activity in the scene, where small motions may not have been captured. Future work may seek to semi-automatically label the data for motion tracking using, for example, the SAM 2^TM^ promptable video segmentation AI model.^28^ In addition, data were only acquired at two sites, and variation in the inter-site analysis results reflects this. To provide a robust algorithm, data from more sites should be used for development, where comprehensive sets for training, testing and validation can be used during its development. This is, however, an expensive undertaking. The use of data augmentation techniques may serve to minimize this by providing additional synthetic data. For example, a virtual digital twin could be created using a 3D synthetic model development software package such as Blender^TM^ (https://www.blender.org/). The algorithm currently does not run fast enough to compute the activity in real-time on a standard PC, rather it requires off-line analysis. However, a higher spec computer or custom-made hardware could address this in future versions of the system.

## Conclusion

We have demonstrated the viability of continuous non-contact monitoring of neonatal activity using a depth sensing camera system. Accurate motion detection correlating to the vital signs is feasible using depth cameras at various gestational ages and weights, for neonates in open/closed incubators, cribs or bassinets. Further studies are required to determine associations with the measured activity and pertinent clinical outcomes.

## Supplementary information

SUPPLEMENTARY MATERIAL -2

Supplementary Material

## Data Availability

The datasets generated during and/or analyzed during the current study are not publicly available as the source data contains identifiable video of the participant volunteers and cannot be shared in order to protect study participant privacy, but may be available from the corresponding author on reasonable request
