# Supplementary material for: Touchless monitoring of neonatal activity: a multi-center study
Source: Pediatr Res. 2025 Jul 24;99(1):96–105. doi: 10.1038/s41390-025-04294-5 (PMC12920080; doi:10.1038/s41390-025-04294-5)
Supplement: Supplementary file 1 — SUPPLEMENTARY MATERIAL -2 [file 41390_2025_4294_MOESM1_ESM.pdf]

Supplementary Material

Touchless Monitoring of Neonatal Activity: A Multi-Center Study

Paul S Addison, Mridula Gunturi, Dean Montgomery
Research and Development, Acute Care & Monitoring, Medtronic, Technopole Centre, Edinburgh, UK.
Rangasamy Ramanathan, Manoj A Biniwale
Department of Pediatrics, Cedars Sinai Guerin Children's, Cedars Sinai Medical Center, Los Angeles, CA, USA.
Dale Gerstmann, Jeffrey Clemmer, Rena Nelson
3 Timpanogos Regional Hospital, Orem, Utah, USA.

Site Details

NICU at Timpanogos, Orem, Utah

- **NICU level of care:** Level III, inborn newborns and regional area neonatal transports
- **bed capacity:** 24
- **patient acuity:** 23 weeks and above; body cooling for HIE infants 35 weeks and older
- **standard monitoring practices:** continuous non-invasive skin temp, SpO2, RR, BP, HR, HRV (HeRO); aEEG, invasive BP and body temp as needed

NICU at Los Angeles General Medical Center, Los Angeles, CA

- **NICU level of care:** Level III
- bed capacity: 39
- patient acuity: All levels of care provided, including surgery, inhaled nitric oxide Rx, etc.
- standard monitoring practices: All patients in the NICU are on continuous monitoring of heart rate, respiratory rate, blood pressure (intermittently or continuously) and SPO2 with pulse ox.

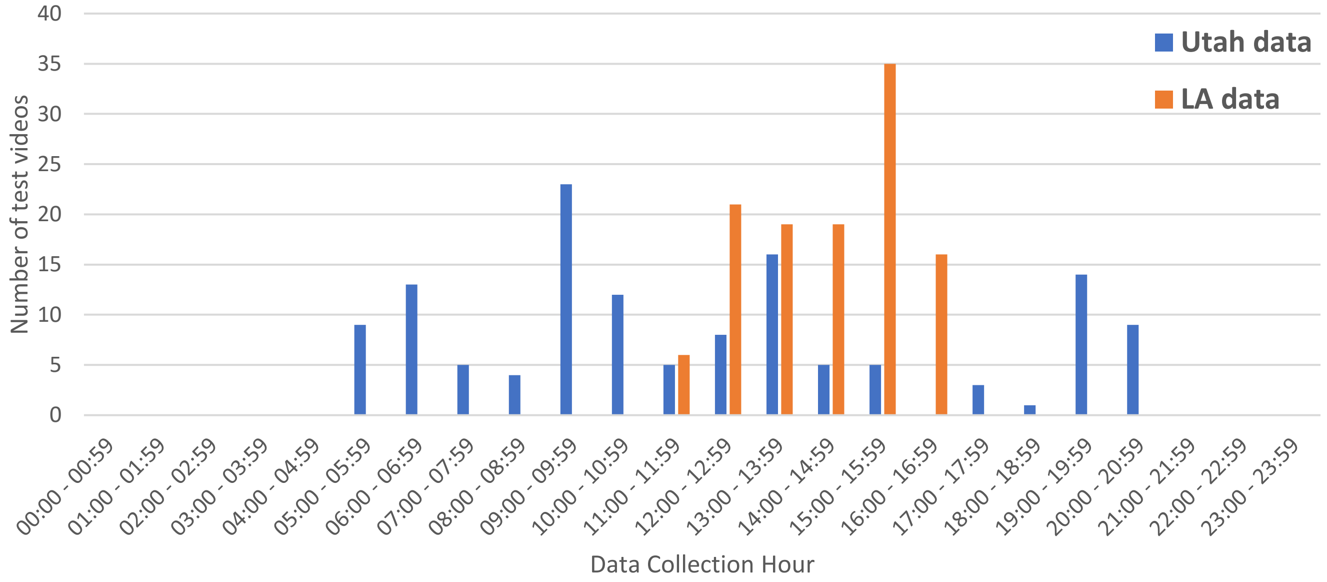

Figure S1– Periods During the Day when Video Data was Collected

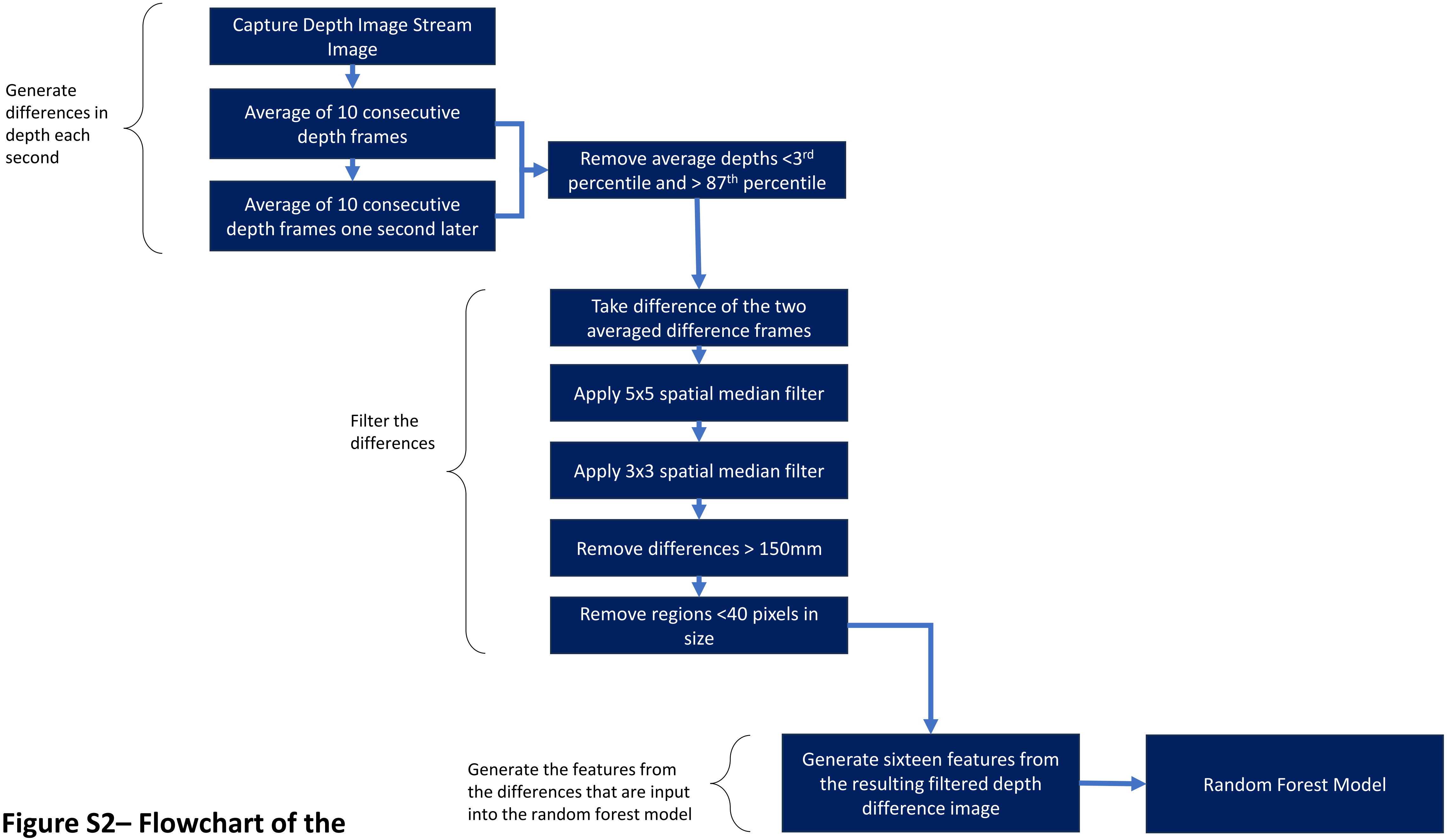

Figure S2– Flowchart of the Data Pre-processing Steps

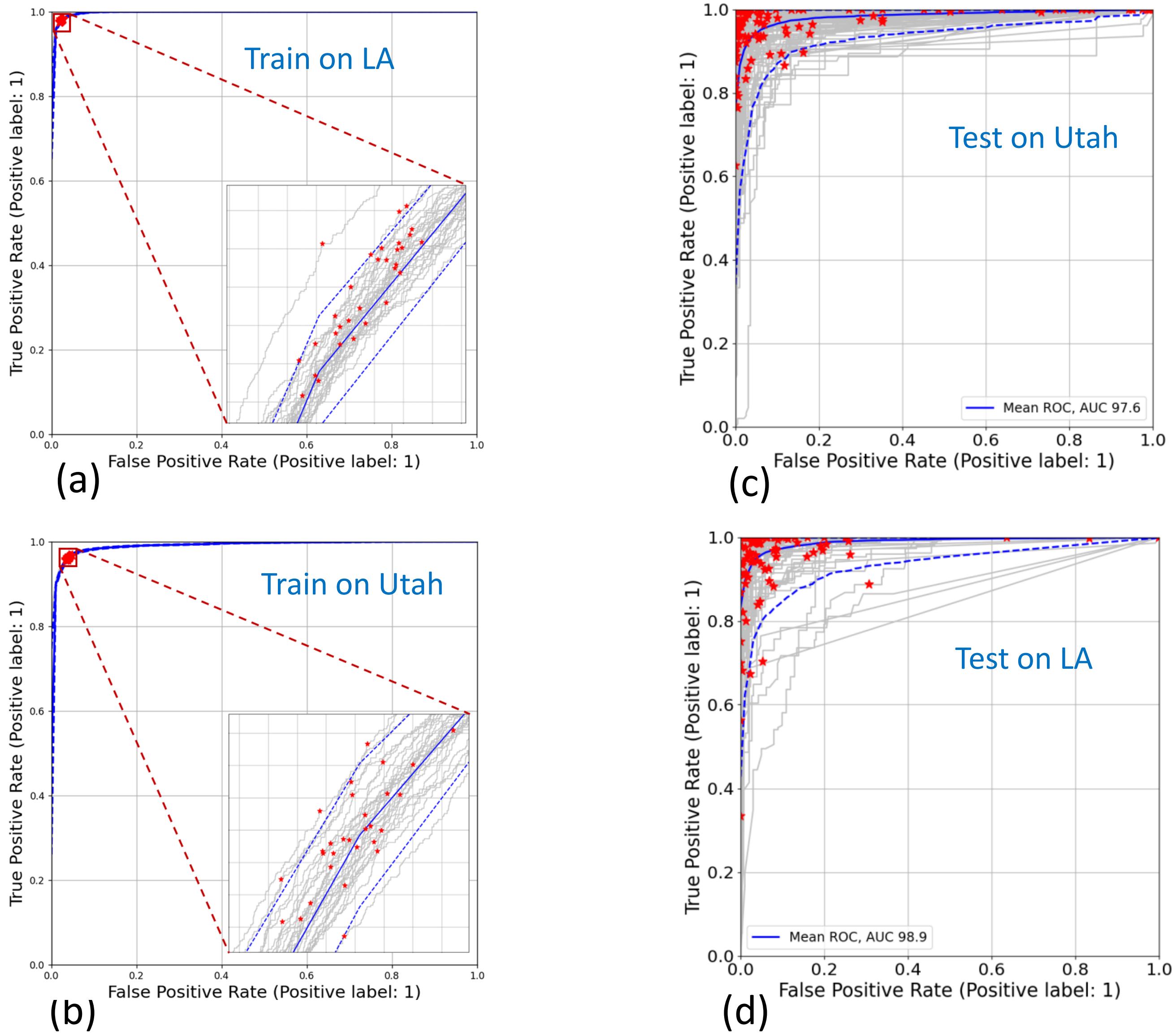

Figure S3: Training and Testing on a Per-Site Basis

The optimal points are indicated on each of the ROC curves by a red asterix.

The mean is shown as a solid blue line and the confidence intervals as dashed blue lines.

Medical Condition	Utah Count	LA Count	Total Count
Respiratory Distress/ Failure/ Depression/ Insufficiency	16	14	30
Feeding problems	8	1	9
Low Birth Weight	4	2	6
Hypoglycemia	1	2	3
R/O Sepsis	3	0	3
Apnea of Prematurity	3	0	3
Other	9	6	15

Table S1 – Table of Medical Conditions reported in the Case

Note that some neonates had more than a single medical condition noted.

Feature Number	Feature Description
**1**	Number of pixels in the depth difference frame where the depth difference is > 3mm
**2**	Number of pixels in the depth difference frame where the depth difference is >3mm and <= 7mm
**3**	Number of pixels in the depth difference frame where the depth difference is > 7mm
**4**	Number of pixels in the depth difference frame where the depth difference is > 1mm and <= 3mm, divided by the total number of valid (non-null and non-zero) pixels in the depth difference frame
**5**	Number of pixels in the depth difference frame where the depth difference is > 3mm and <= 7mm, divided by the total number of valid (non-null and non-zero) pixels in the depth difference frame
**6**	Sum of pixels in the depth difference frame where the depth difference is > 3mm
**7**	Sum of pixels in the depth difference frame where the depth difference is > 1mm and <= 3mm
**8**	Sum of pixels in the depth difference frame where the depth difference is > 7mm
**9**	Sum of pixels in the depth difference frame where the depth difference is > 3mm and <= 7mm
**10**	Mean of all valid (non-null and non-zero) pixels in the depth frame where the corresponding pixels in the depth difference frame have values > 3mm
**11**	Mean of all valid (non-null and non-zero) pixels in the depth frame where the corresponding pixels in the depth difference frame have values > 1mm and <= 3mm
**12**	Mean of all valid (non-null and non-zero) pixels in the depth frame where the corresponding pixels in the depth difference frame have values > 7mm
**13**	Mean of all valid (non-null and non-zero) pixels in the depth frame where the corresponding pixels in the depth difference frame have values < 3mm and <= 7mm
**14**	Sum of valid (non-null and non-zero) pixels in the standard deviation frame (frame of per-pixel standard deviation over the first 10 and last 10 depth frames in every second) where the corresponding depths are < the 75th percentile of all depths greater than 200mm, divided by the sum of all valid (non-null and non-zero) pixels in the standard deviation frame
**15**	Kurtosis over all non-null and nonzero pixels in the depth frame
**16**	Mean of pixels in the depth frame where depth is < the 75th percentile of all depths greater than 200mm

Table S2 – Table of Input Features

Parameter	Value	Description	Optimization values
Number of temporally-averaged depth frames	10 frames	The number of depth frames that are averaged over time to compute one depth difference frame. It was visually determined that the minimum duration of neonatal motion is approximately 1/3 seconds; given that the frame rate is 30 fps, averaging over 10 frames was considered optimal to reduce non-motion noise in the data.	5, 10
Spatial median filter sizes	5x5, 3x3 pixels	The size of the median-averaging spatial filters that are applied across the frame to reduce noise. Using a larger 5x5 filter first reduces broader speckle noise around edges in the image- for example, around the neonate in the frame. Applying the smaller 3x3 filter provides further smoothing.	0x0 – 5x5, for both filters
Upper depth percentile limit	87%	Upper percentile limit, applied such that pixels with depths above this limit are set to zero. It was observed that most non-motion noise in the depth data occurred at higher depths. To minimize the effect of noise in the input features, it is necessary to apply an upper depth percentile limit.	80% - 97%
Lower depth percentile limit	3%	Lower percentile limit, applied such that pixels with depths below this limit are set to zero. It was observed that a small amount of non-motion noise occurs at lower depths. It is thus necessary to apply a lower depth percentile limit.	0% - 13%
Minimum connected pixel area size	40 pixels	The minimum area of connected nonzero pixels that can be present in the depth difference frame; if the area is lower than 40, all pixels in the connected area are set to zero. This parameter removes very small connected pixel areas of depth difference that are too small to be considered valid neonatal motion, given the range of camera distances and heights of preterm and term neonates.	30, 40, 80, 120

Table S3: Denoising Parameters

Hyperparameter	Value	Overfitting mitigation
Number of trees in ensemble	20	In a Random Forest, the output is calculated by averaging predictions of all decision trees in the ensemble. A higher number of trees reduces the impact of individual trees overfitting, however the improvement in performance is minimal as the number of trees is increased beyond a point.
Maximum tree depth	8	Higher max tree depth of individual trees enables them to capture more complex features- however, excessively deep trees are more likely to overfit. Reducing the max tree depth of individual decision trees mitigates overfitting.
Minimum number of samples required to split node	30	Setting a lower limit on the samples required to split a decision tree node prevents the creation of branches with very few samples- this stops the tree from learning unnecessary detail in the train data and thus mitigates overfitting
Minimum number of samples at a leaf node	5	A leaf node is a decision tree node that is the end of a branch/ has no child nodes. By setting a lower limit on the samples that can be present at leaf nodes, we ensure the tree does not capture noise in the train data- this reduces overfitting.

Table S4: Model Hyperparameters

Feature	Importance<br>mean (std. dev.)
1 to 3mm bin fraction	0.003 (0.001)
**>3mm bin**	**0.215 (0.006)**
>3mm sum	0.007 (0.002)
**1 to 3mm sum**	**0.440 (0.005)**
Average depths > 3mm	0.031 (0.011)
Average depths 1-3mm	0.035 (0.003)
3 to 7mm bin fraction	0.002 (0.004)
**3 to 7mm bin**	**0.163 (0.004)**
>7mm bin	0.000123 (0.000054)
>7mm sum	0.000234 (0.000061)
3 to 7mm sum	0.042 (0.002)
Average depths >7mm	0.000203 (0.000048)
Average depths 3-7mm	0.013 (0.01)
75th percentile std. depths fraction	0.031 (0.002)
Kurtosis of depths	0.01 (0.001)
75th percentile average depths	0.007 (0.001)

(a) Combined Analysis, both sites LOOCV

Feature	Importance
1 to 3mm bin fraction	0.006
**>3mm bin**	**0.217**
>3mm sum	0.003
**1 to 3mm sum**	**0.496**
Average depths > 3mm	0.04
Average depths 1-3mm	0.006
3 to 7mm bin fraction	0.003
**3 to 7mm bin**	**0.169**
>7mm bin	0.000237
>7mm sum	0.000172
3 to 7mm sum	0.044
Average depths >7mm	8.1e-5
Average depths 3-7mm	0.0011
75th percentile std. depths fraction	0.0045
Kurtosis of depths	0.0056
75th percentile average depths	0.0034

(b)Train on LA, test on Utah

Feature	Importance
**1 to 3mm bin fraction**	0.003
>3mm bin	**0.210**
>3mm sum	0.007
**1 to 3mm sum**	**0.432**
Average depths > 3mm	0.008
Average depths 1-3mm	0.039
3 to 7mm bin fraction	0.001
**3 to 7mm bin**	**0.168**
>7mm bin	0.000147
>7mm sum	0.029
3 to 7mm sum	0.044
Average depths >7mm	0.000223
Average depths 3-7mm	0.009
75th percentile std. depths fraction	0.030
Kurtosis of depths	0.009
75th percentile average depths	0.009

(c) Train on Utah, test on LA

Table S5: Feature Importance

The top three most important features are in bold. Although these have much higher mean feature importance, the remaining features are also required to classify edge cases. Using only the top three features results in poorer performance.

Camera Position	Mean Distance (mm)	Utah count	LA count	Total count	AUC Mean	AUC 95% Conf. Interval	SENS Mean	SENS 95% Conf. Interval	SPEC Mean	SPEC 95% Conf. Interval	YI	YI 95% Conf. Interval	p-value
1	448	32	19	51	0.98	(0.98, 0.99)	0.96	(0.94, 0.97)	0.93	(0.91, 0.96)	0.89	(0.86, 0.92)	0.47
2	641	17	35	52	0.99	(0.99, 1.00)	0.94	(0.91, 0.96)	0.93	(0.89, 0.96)	0.87	(0.83, 0.91)	
3	455	24	22	46	0.98	(0.96, 1.00)	0.95	(0.90, 0.99)	0.91	(0.85, 0.98)	0.86	(0.78, 0.93)	
4	626	16	23	39	0.99	(0.97, 1.00)	0.93	(0.88, 0.98)	0.91	(0.84, 0.98)	0.83	(0.76, 0.91)	
5	465	14	2	16	0.99	(0.98, 1.00)	0.94	(0.90, 0.98)	0.96	(0.94, 0.98)	0.90	(0.86, 0.94)	
6	661	29	15	44	0.97	(0.95, 1.00)	0.92	(0.87, 0.97)	0.91	(0.85, 0.97)	0.83	(0.75, 0.90)	

Gestational Age (weeks)	Mean Age (weeks)	Utah count	LA count	Total count	AUC Mean	AUC 95% Conf. Interval	SENS Mean	SENS 95% Conf. Interval	SPEC Mean	SPEC 95% Conf. Interval	YI	YI 95% Conf. Interval	p-value
26 – 28	27.54	14	9	23	0.99	(0.98, 1.00)	0.95	(0.92, 0.98)	0.94	(0.91, 0.98)	0.89	(0.84, 0.94)	0.67
28 – 32	30.93	31	18	49	0.98	(0.96, 0.99)	0.92	(0.89, 0.95)	0.95	(0.92, 0.98)	0.87	(0.83, 0.91)	
32 – 36	34.45	43	30	73	0.98	(0.96, 1.00)	0.93	(0.89, 0.97)	0.91	(0.87, 0.95)	0.84	(0.79, 0.89)	
> 36	37.69	45	58	103	0.99	(0.99, 1.00)	0.95	(0.93, 0.97)	0.91	(0.87, 0.95)	0.86	(0.82, 0.90)	

Weight (Kg)	Mean Weight (Kg)	Utah count	LA count	Total count	AUC Mean	AUC 95% Conf. Interval	SENS Mean	SENS 95% Conf. Interval	SPEC Mean	SPEC 95% Conf. Interval	YI	YI 95% Conf. Interval	p-value
0 – 1	0.95	0	6	6	0.99	(0.97, 1.00)	0.90	(0.83, 0.97)	0.96	(0.87, 1.00)	0.86	(0.74, 0.99)	0.48
1 – 2	1.73	39	30	69	0.99	(0.98, 0.99)	0.95	(0.94, 0.97)	0.94	(0.94, 0.95)	0.90	(0.87, 0.92)	
2 – 3	2.54	60	64	124	0.98	(0.97, 0.99)	0.93	(0.90, 0.96)	0.93	(0.90, 0.96)	0.86	(0.82, 0.89)	
3 – 4	3.45	33	16	49	0.98	(0.97, 0.99)	0.94	(0.92, 0.97)	0.87	(0.79, 0.94)	0.81	(0.74, 0.88)	

Bed Type	n/a	Utah count	LA count	Total count	AUC Mean	AUC 95% Conf. Interval	SENS Mean	SENS 95% Conf. Interval	SPEC Mean	SPEC 95% Conf. Interval	YI	YI 95% Conf. Interval	p-value
Bassinette	n/a	70	22	92	0.99	(0.98, 0.99)	0.95	(0.93, 0.97)	0.90	(0.86, 0.94)	0.85	(0.80, 0.89)	0.14
Crib	n/a	0	28	28	0.99	(0.99, 1.00)	0.94	(0.93, 0.96)	0.97	(0.93, 1.00)	0.91	(0.86, 0.96)	
Closed isolette	n/a	19	66	85	0.99	(0.98, 1.00)	0.96	(0.91, 1.00)	0.91	(0.90, 0.93)	0.87	(0.83, 0.91)	
Open isolette	n/a	43	0	43	0.96	(0.93, 1.00)	0.88	(0.82, 0.95)	0.96	(0.93, 0.98)	0.84	(0.77, 0.90)	

Table S6: Results of the Subgroup Analyses

	Combined Analysis: LOOCV, both sites		Inter-Site Analysis: Train on LA, test on Utah		Inter-Site Analysis: Train on Utah, test on LA		Intra-Site Analysis: LOOCV, LA data only		Intra-Site Analysis: LOOCV, Utah data only	
**Result Statistic**	**Mean**	**95% conf. interval, mean**	**Mean**	**95% conf. interval, mean**	**Mean**	**95% conf. interval, mean**	**Mean**	**95% conf. interval, mean**	**Mean**	**95% conf. interval, mean**
Sensitivity, train	96.3	(96.2, 96.3)	99.8	*	99.2	*	98.0	(97.9, 98.1)	96.2	(96.1, 96.3)
Specificity, train	96.7	(96.7, 96.8)	98.3	*	96.6	*	97.7	(97.6, 97.7)	96.0	(95.9, 96.1)
ROC-AUC, train	99.4	(99.4, 99.4) **	97.3	*	95.6	*	99.8	(99.8, 99.8) **	99.2	(99.2, 99.2) **
Sensitivity, test	93.8	(92.3, 95.3)	94.2	(92.0, 96.5)	95.1	(93.3, 96.9)	96.4	(95.1, 97.7)	92.1	(89.7, 94.6)
Specificity, test	92.2	(90.0, 94.3)	81.5	(76.2, 86.7)	91.9	(88.9, 94.9)	93.6	(90.6, 96.5)	91.8	(89.4, 94.3)
ROC-AUC, test	98.4	(97.8, 99.0)	97.6	(96.5, 98.7)	98.9	(98.5, 99.4)	99.2	(98.7, 99.7)	97.4	(96.2, 98.6)

* There is only a single ROC curve generated from the training of all the data from a single site (before testing on the individual cases of the test site), hence no confidence intervals are reported.

** All reported means and 95% confidence interval limits are rounded to three significant figures for convenience.

Table S7: All results tabulated for comparison
